# Case Report: Mevalonic Aciduria Complicated by Acute Myeloid Leukemia After Hematopoietic Stem Cell Transplantation

**DOI:** 10.3389/fimmu.2021.782780

**Published:** 2021-12-07

**Authors:** Hyery Kim, Beom Hee Lee, Hyo-Sang Do, Gu-Hwan Kim, Sunghan Kang, Kyung-Nam Koh, Ho Joon Im

**Affiliations:** ^1^ Division of Pediatric Hematology/Oncology, Department of Pediatrics, Asan Medical Center Children’s Hospital, University of Ulsan College of Medicine, Seoul, South Korea; ^2^ Department of Pediatrics, Asan Medical Center Children’s Hospital, University of Ulsan College of Medicine, Seoul, South Korea; ^3^ Medical Genetics Center, Asan Medical Center, University of Ulsan College of Medicine, Seoul, South Korea; ^4^ Asan Institute for Life Sciences, Asan Medical Center, University of Ulsan College of Medicine, Seoul, South Korea

**Keywords:** mevalonic aciduria, mavalonic kinase deficiency, hematopoietic stem cell transplantation, acute myeloid leukemia, inflammation

## Abstract

Mevalonic aciduria (MA) is the most severe clinical subtype of mevalonate kinase deficiency (MKD) caused by an inherited defect in the mevalonate pathway. The treatment of MKD focuses on the suppression of recurrent hyperinflammatory attacks using anti-inflammatory drugs. Recently, allogeneic hematopoietic stem cell transplantation (HCT) was shown to successfully ameliorate autoinflammatory attacks in patients with MKD. Here, we report a case of an infant who showed severe recurrent systemic inflammation and was diagnosed with MA. Although she responded to steroids, her symptoms relapsed after the dose was tapered, and organ deterioration occurred. Therefore, at the age of 11 months, HCT from a matched, unrelated donor was performed for curative treatment. However, at 50 days after transplantation, acute myeloid leukemia was diagnosed, which was chemo-refractory. A second HCT from her haploidentical father was performed to treat the acute myeloid leukemia, but the patient died of sepsis on day 4 after transplantation. This is the first report of malignancy following HCT for MA. Our findings suggest that normalizing the mevalonate pathway after HCT in patients with MKD impacts patients differently depending on the clinical spectrum and severity of disease.

## Introduction

Mevalonate kinase deficiency (MKD) involves a spectrum of rare autoinflammatory disorders caused by an inherited defect in the isoprenoid biosynthetic pathway (1). There are two disease entities of MKD: mevalonic aciduria (MA) and hyperimmunoglobulinemia D, and periodic fever syndrome. These two clinical phenotypes have been linked with a shared genetic lesion in *MVK* (mevalonate kinase) on chromosome 12q24 (2). Pathogenic variants in this gene result in dysfunction in the mevalonate pathway and cause episodes of hyperinflammation with increased secretion of IL-1beta. MA is the most severe clinical subtype of MKD and presents in the first few months of life, with antenatal presentation associated with a high rate of stillbirth in affected families (3). Clinical symptoms of affected patients include a severe failure to thrive, and fatal autoinflammatory attacks with fever, mucoid and cutaneous lesions, abdominal pain, arthralgias, hepatosplenomegaly, and liver dysfunction.

Treatment of MA is focused on suppressing recurrent hyperinflammatory attacks to prevent organ deterioration. Nonsteroidal anti-inflammatory drugs and corticosteroids are the most commonly used drugs. In addition, biologic agents targeting IL-1 or IL-6 as the main cytokines in the inflammatory process in MA are used. Allogeneic stem cell transplantation (HCT) is an alternative approach for treating patients with MKD and has successfully ameliorated autoinflammatory attacks (4, 5). We report a patient with refractory MA who developed acute myeloid leukemia (AML) immediately after receiving allogeneic HCT.

## Case Description

A girl weighing 2.49 kg was delivered at the gestational age of 37 months *via* cesarean section because of fetal distress. She was the first child of the family, and the parents were healthy without specific diagnosed disease. She was transferred to the neonatal intensive care unit because of respiratory distress. Hepatosplenomegaly was detected, and initial laboratory tests revealed the following: white blood cell count, 23,650/μL; hemoglobin, 18 g/dL; platelet count, 158,000/μL; aspartate aminotransferase, 741 IU/L; alanine aminotransferase, 324 IU/L; C-reactive protein (CRP), 19.72 mg/dL; total bilirubin 11 mg/dL; direct bilirubin 7.2 mg/dL; and gamma-glutamyltransferase, 118 U/L. There was no evidence of infection or inborn error of metabolism, as observed by tandem mass screening. A liver biopsy showed portal and panlobular infiltration of mixed inflammatory cells without specific diagnostic findings. All other tests showed nonspecific findings except anti-smith antibody and cytoplasmin antibody tests. Based on the suspected diagnosis of autoimmune hepatitis with persistent cholestasis and elevated liver enzyme levels, treatment with 2 mg/kg/day methylprednisolone was started. Her levels of bilirubin and liver enzymes gradually decreased over 6 weeks of steroid administration. However, fever developed repeatedly when the steroid dosages were reduced. The fever was completely resolved only after re-escalation of steroids. She was discharged and prescribed oral steroids at 2 mg/kg/day.

She was readmitted to the hospital at the age of 4 months because of a spiking fever. Her laboratory tests revealed the following: white blood cell count, 42,100/μL; hemoglobin, 7.7 g/dL; platelet count, 311,000/μL; aspartate aminotransferase, 127 IU/L; alanine aminotransferase, 227 IU/L; CRP, 3.71 mg/dL; erythrocyte sedimentation rate, 47 mm/h; and total bilirubin 1.2 mg/dL. Computed tomography showed right paraspinal soft tissue with mass massive hepatosplenomegaly. Therefore, malignancy was suspected, and biopsy of the mass and bone marrow was performed. The karyotype was 46, XX. The mass biopsy showed benign reactive lymphoid hyperplasia, with no malignant cells in the bone marrow, although myelofibrosis with dyserythropoietic anemia was noted.

**Figure 1:**
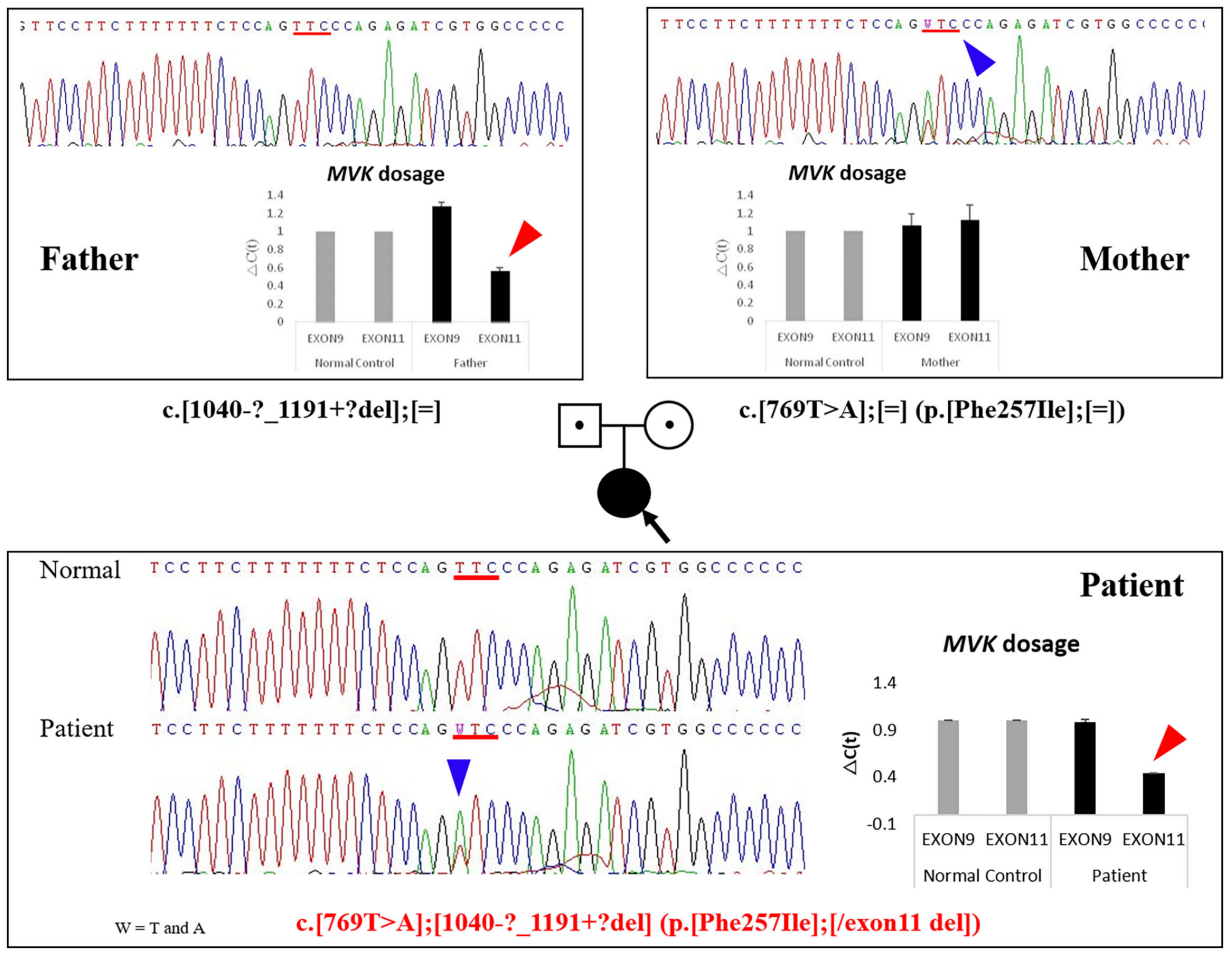
Identification of mutations by DNA sequencing and relative quantification of *MVK* exon 9 and 11 expression in the patient and her parents. The heterozygous *MVK* mutation c.769T>A (p.Phe257Ile) in the patient was inherited from her mother (blue arrows), and the exon 11 deletion was inherited from her father (red arrows). Fold-change in expression of MVK exons 9 and 11 measured using quantitative RT-PCR. GAPDH expression was used to normalize target gene expression. The relative expression of each gene was calculated as the log2 of 2^-△Ct^ values. *MVK*, mevalonate kinase; RT-PCR, reverse transcription PCR; *GAPDH*, glyceraldehyde 3-phosphate dehydrogenase.

**Figure 2:**
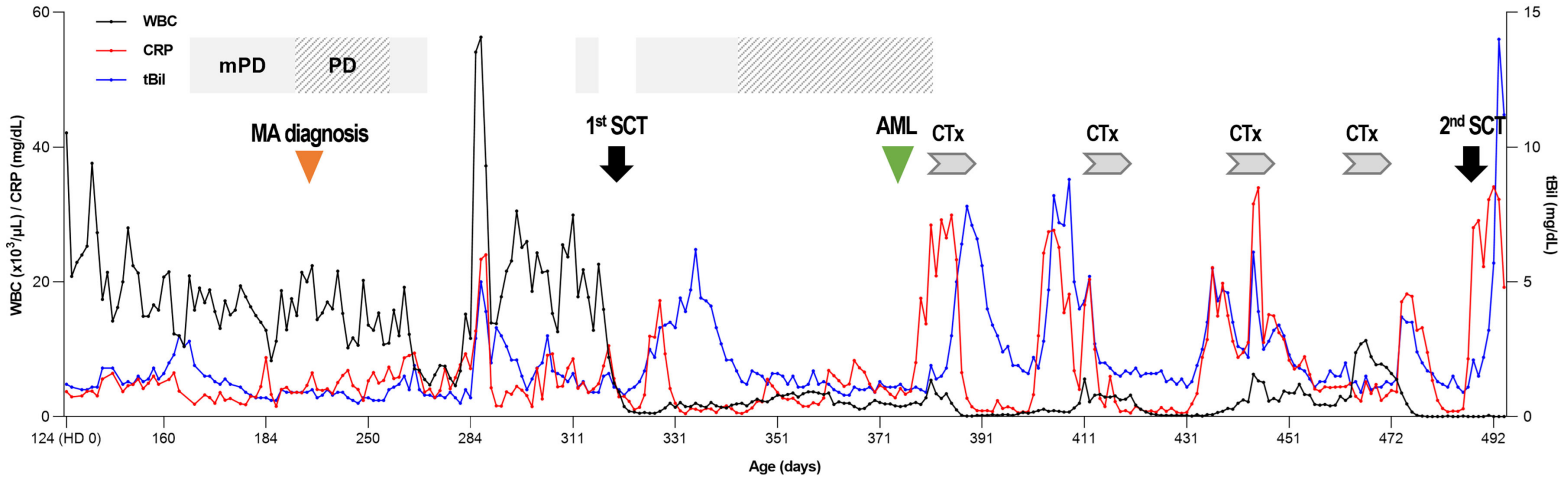
Clinical course of the patient. At the time of diagnosis, leukocytosis was prominent, and CRP was above the normal range. After the 1^st^ HCT, the WBC count was normalized, and CRP and total bilirubin levels decreased to a normal range and showed alternate increases and decreases during chemotherapy. WBC, white blood cell; CRP, C-reactive protein; tBil, total bilirubin; mPD, methylprednisolone; PD, prednisolone; MA, mevalonic aciduria; HCT, hematopoietic stem cell transplantation; AML, acute myeloid leukemia; CTx, chemotherapy.

Under the suspicion of congenital disease that causes systemic inflammation, whole-exome sequencing (WES, Methods in Supplementary Material) was performed, which revealed that the patient carried a heterozygous mutation of c.769T>A (p.Phe257Ile) in *MVK* (**Table 1**; **Figure 1**; **Methods in Supplementary Material**). In the result of WES, heterozygous deletionat exon11 of *MVK* gene was suspected with lower depth than other exons.A real-time polymerase chain reaction (PCR) assay was performed to analyze the expression of *MVK* in the patient and parents, which revealed decreased expression of exon 11 in the patient and father (**Figure 1** and **Methods in Supplementary Material**). The mevalonic acid and mevalonic lactone concentrations in the urine were increased to 2 mmol/mol of creatinine (reference, 0 mmol/mol of creatinine) and 497.3 mmol/mol of creatinine (reference, 0 mmol/mol of creatinine), respectively. Based on these results, the patient was finally diagnosed with MA at the age of 6 months=. Her fever and levels of inflammatory markers began decreasing in response to intravenous methylprednisolone at 30 mg/kg/day for 3 days but reappeared repeatedly when the drug was tapered or changed to oral prednisolone (**Figure 2**). She was unable to discontinue steroids because of recurrent inflammatory fever; however, candidiasis persisted, and mild coronary artery dilatation was detected because of repeated episodes of severe systemic inflammation. Therefore, we decided to perform HCT as a definite treatment.

**Table 1:** Results of *in silico* analysis for the candidate mutation in the patient.

Gene	*MVK*
Variant type	amino acid substitution variant
HGVS coding sequence name	NM_000431.2:c.769T>A
HGVS protein sequence name	p.Phe257Ile
***FUNCTIONAL PREDICTIONS***	
SIFT	Tolerated, 0.10
Polyphen2_HDIV	Probably damaging, 0.995
MutationTaster	Disease causing
MutationAssessor	Medium, 3.24
FATHMM	Damaging, -3.2
PROVEAN	Deleterious, -3.673
MetaSVM	Deleterious, 0.857
MetaLR	Deleterious, 0.856
M-CAP	Deleterious, 0.511
CADD	24.3
DANN	0.982
fathmm-MKL	Deleterious, 0.886
***CONSERVATION SCORES***	
GERP++	4.6
phyloP20way	1.061
phastCons20way	0.901
SiPhy_29way_logOdds	10.291

The conditioning regimen consisted of busulfan (4.4 mg/kg/day, d-5~d-4), fludarabine (1 mg/kg/day, d-8~d-4), cyclophosphamide (50 mg/kg/day, d-3~d-2), and rabbit anti-thymocyte globulin (ATG, 2.5 mg/kg/day, d-8~d-6). Prophylaxis for graft-versus-host disease consisted of cyclosporine and mycophenolate mofetil. The patient received 13.84 × 10^6^ CD34 cells/kg from an unrelated human leukocyte antigen-matched donor. Peripheral blood stem cells were collected as described previously (6). She experienced mild sinusoidal obstruction syndrome but was otherwise relatively uneventful until neutrophil engraftment at day 27. Her laboratory tests at day 27 revealed the following: white blood cell count, 2,000/μL; hemoglobin, 9.0 g/dL; platelet count, 29,000/μL; neutrophil count, 1,170/μL. A chimerism test at day 21 showed 94% of donor-derived cells, and urinary mevalonic acid and mevalonic lactone levels were decreased to the normal values of 0.3 and 80.4 mmol/mol of creatinine, respectively.

**Figure 3:**
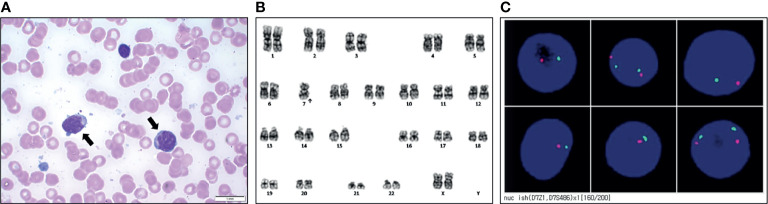
Results of work-up at the diagnosis of acute myeloid leukemia. **(A)** Bone marrow smears taken from the patient (myeloblast: black arrows). **(B)** Representative karyotype of the blast was 45, XX, -7. **(C)** Fluorescence *in situ* hybridization of the 7q deletion showing monosomy 7.

Cytokine evaluation was performed using the Millipore Human Cytokine/Chemokine Magnetic Bead Panel (Merck KGaA Inc., Darmstadt, Germany), and the levels of cytokines such as Il-1β, IL-6, and TNFα, which are involved in systemic inflammation, were found to be decreased remarkably (**Figure 3**). However, recipient chimerism increased by up to 34% at day 42; therefore, the immunosuppressant was stopped, and a donor lymphocyte infusion was performed. At day 50, the chimerism test showed 91% recipient cells; a bone marrow biopsy was conducted owing to the persistent abnormal chimerism status and pancytopenia (white blood cell count, 1,500/μL; hemoglobin, 9.3 g/dL; platelet count, 20,000/μL; neutrophil count, 340/μL). Results from the bone marrow biopsy showed hypercellularity of the bone marrow tissue, with an increase in the number of myeloblasts (**Figure 3A**). Immunophenotyping of bone marrow cells showed 20.6% myeloblasts expressing CD34 and CD117, but lacking CD3 and CD20, confirming AML with M2. Trilineage dysplasia was unremarkable, whereas cytogenetic analysis revealed a monosomic karyotype, 45,XX,-7[20] (**Figure 3B**). Fluorescence *in situ* hybridization (FISH) revealed 7q deletion in myeloblasts, showing monosomy 7 (**Figure 3C**).

As an induction chemotherapy for AML, modified A-triple-V was initiated at day 65 after the 1^st^ HCT (7). However, the patient’s leukemia persisted even after two additional reinduction chemotherapy steps with MRC AML12 (8) and a second cycle of modified A-triple-V. Because the AML was refractory, a second HCT was planned with the father as the haploidentical donor following pre-HCT clofarabine with cytarabine treatments. Clofarabine (30 mg/m^2^/day) and cytarabine (1 g/m^2^/day) were infused for 5 days, and the conditioning regimen for HCT was initiated after 5 days of rest. Conditioning consisted of low-dose total-body irradiation (2 Gy/day, d-7~d-6), fludarabine (1 mg/kg/day, d-7~d-3), cyclophosphamide (50 mg/kg/day, d-4~d-3), and rabbit anti-thymocyte globulin (2 mg/kg/day at d-2, 1 mg/kg/day at d-1) (9). The αβ^+^ T cells were depleted by negative selection using the CliniMACS system (Miltenyi-BioTec). The αβ T-cell depleted graft was composed of 9.17 × 10^6^ CD34 cells/kg, 4.96 × 10^4^ αβ T-cell cells/kg, and 2.54 × 10^7^ γδ T-cell cells/kg, which were infused without event. On day 1, rituximab infusion was used to deplete B cells at a dose of 375 mg/m^2^. Supportive care and post-transplant assessments were conducted as we described previously (10). However, her total bilirubin and CRP levels were elevated to 5.7 and 34.09 mg/dL at day 2, respectively, and fever and hypotension developed. Inotropes and additional broad-spectrum antibiotics were initiated under the assumption of septic shock. However, her vital signs did not recover, and the patient passed away at day 4 of haplo-HCT because of *Acinetobacter baumannii* bacteremia.

## Discussion

Allogeneic HCT has been proposed as a curative treatment for patients with MKD based on the hypothesis that donor-derived mononuclear cells are a source of MVK enzyme. To date, eight patients with severe MKD who received allogeneic HCT have been reported (4, 5, 11–16). A complete response of autoinflammatory disease after HCT was described in all but one patient who experienced a relapse of recurrent fever episodes requiring treatment with an anti-IL1 drug (canakinumab), despite stable full donor engraftment (13).

The patient in our study experienced continuous inflammation-induced fever that evolved to coronary artery dilatation. However, steroids could not be tapered or discontinued, and other anti-inflammatory drugs could not be used because of age-limit regulations. Therefore, we decided to perform allogeneic HCT in this refractory case. However, the patient developed AML immediately after the 1^st^ HCT. To the best of our knowledge, there are no reports of patients with MKD who developed malignancies with or without HCT.

The exact mechanism of leukemogenesis in our case could not be determined. The onset of AML was only within 2 months in our case. In previous cases, the median time to secondary AML or myelodysplastic syndrome (MDS) after HCT was reported as 2.5~3 years, and the earliest case occurred 4 months after HCT (17, 18). Therefore, the short time interval in our case suggested that pre-existing conditions are relevant.

However, MKD is not known as a cancer predisposition syndrome. In addition, no significant pathogenic/likely pathogenic variants among cancer-predisposing genes were found based on OMIM.org and a previous reference (19) in the WES data of this patient.

Considering that the patient was diagnosed with AML with monosomy 7, which is one of the most frequent chromosomal aberrations in myeloid neoplasms regardless of patient age and disease etiology, familial monosomy 7 could be suspected. Familial monosomy 7 defined as bone marrow monosomy 7 has been reported, wherein most patients are either children or adolescents (20). However, there were neither any special chromosomal findings before the diagnosis of AML in this patient nor any medical history of leukemia, myelodysplastic syndrome, or unexplained thrombocytopenia in her family members. Therefore, no definite genetic predisposing variants could explain the occurrence of AML in our patient.

However, the oncogenic activity of the mevalonate pathway may be related to leukemogenesis (21). According to a previous study of chronic lymphocytic leukemia, mevalonate can stimulate the proliferation of primary leukemic cells (22). In another study, culturing breast cancer cells with mevalonate resulted in increased tumor growth (23). Mevalonate metabolism provides farnesyl diphosphate and geranylgeranyl diphosphate, which are the activated forms of farnesyl and geranylgeranyl, respectively (24). Both farnesyl diphosphate and geranylgeranyl diphosphate are essential for post-translational prenylation of small GTP-binding proteins. Many small GTPases, such as Ras and Rho, as members of the Ras superfamily of small GTPases involved in tumorigenesis, must be isoprenylated (25). Therefore, inhibiting the mevalonate pathway can reduce the isoprenylation of small GTPases, thereby inducing cancer cell death (26–28). In contrast, uncontrolled flux of mevalonate pathway metabolites promotes sustained Ras protein activation by constitutive prenylation, triggering malignant transformation (13).

In our case, the mevalonate pathway was inhibited initially; therefore, farnesyl diphosphate and geranylgeranyl diphosphate were suppressed. Early after HCT, the signs of systemic inflammation including cytokine levels were successfully resolved (**Supplementary Figure 1**), and the urinary MA level was decreased. However, a sudden surge in mevalonate metabolite levels may have occurred after MVK replacement by donor grafts *via* HCT, which may have initiated leukemogenesis.

In addition, we reported the second youngest case at the time of HCT (11 months of age) among the eight patients with MKD who underwent HCT. The age of other patients who were successfully treated using HCT were 15 months, 22 months in the most recent report of αβ T-cell-depleted haploidentical HCT (16), and 2–8 years of age (4, 5, 12, 14). The other two patients underwent HCT at 138 days and 14 months of age and passed away from sepsis at 3.5 months post-HCT and showed relapse at 18 months after HCT, respectively. All three of the youngest patients had post-transplant events. Although the severity of clinical symptoms in each patient could not be compared, transplantation at a younger age may represent more severe clinical spectra or multiorgan involvement. When HCT was performed in younger patients with more severe clinical spectra, the discrepancy of mevalonate pathway activation between pre- and post-HCT would be larger than that in older patients with less severe clinical spectra. The youngest patient among well-treated patients reported to date is a 15-month-old female who underwent αβ T-cell depleted haploidentical HCT. The transplantation course was uneventful but she showed high levels of urine MA after transplantation (16). This indicates that a minimum correction occurred in this patient to stabilize systemic inflammation. Thus, mevalonate pathway metabolism was functional and resulted in adequate amounts of mevalonate metabolites as well as prevention of immune disruption or malignant transformation.

## Conclusion

This is the first report of a case of malignancy following HCT for MA. Our findings suggest that HCT does not always cure MA, in contrast to prior reports, and transplantation may be complicated by malignancy. Normalization of the mevalonate pathway after HCT in patients with MKD may impact patients differently depending on the clinical spectra and severity of the disease. The influence of the correlation between the genetic phenotype and clinical subtype on the outcome of HCT should be explored in further studies.

## Patient Perspective

The parents of the patient were fully engaged throughout the treatment process. We had multiple in-depth conversations regarding the potential risks and benefits of HCT and chemotherapy. After the patient passed away, the parents agreed to the publication of this case report in the hope of furthering medical knowledge in this area. The Institutional Review Board of the Asan Medical Center (Seoul, Korea) approved the protocol of this report (IRB No. S2021-2408-0001). Informed consent was waived by the Institutional Review Board.

## Data Availability Statement

The raw data supporting the conclusions of this article will be made available by the authors, without undue reservation.

## Ethics Statement

The studies involving human participants were reviewed and approved by The Institutional Review Board of Asan Medical Center (Seoul, Korea) approved the protocol of this report (IRB No. S2021-2408-0001). Written informed consent for participation was not provided by the participants’ legal guardians/next of kin because: Informed consent was waived by the Institutional Review Board.

## Author Contributions

HK and BL conceived the study and defined the concept. HK and BL collected and interpreted the data. H-SD conducted the cytokine assay. G-HK prepared figures and supplementary material, HK wrote the initial draft of the manuscript. BL, SK, K-NK, and HI critically discussed the data, revised the manuscript for intellectual content. All authors agreed to be accountable for all aspects of the work in ensuring that questions relating to the accuracy or integrity of any part of the work were appropriately investigated and resolved. All authors contributed to the article and approved the submitted version.

## Funding

This work was supported by a National Research Foundation of Korea (NRF) grant, funded by the Korean government (MSIT) (No. NRF-2018M3A9H1078335 and NRF-2021R1A2C1095874).

## Conflict of Interest

The authors declare that the research was conducted in the absence of any commercial or financial relationships that could be construed as a potential conflict of interest.

## Publisher’s Note

All claims expressed in this article are solely those of the authors and do not necessarily represent those of their affiliated organizations, or those of the publisher, the editors and the reviewers. Any product that may be evaluated in this article, or claim that may be made by its manufacturer, is not guaranteed or endorsed by the publisher.
