# Supplementary material for: Case Report: Mevalonic Aciduria Complicated by Acute Myeloid Leukemia After Hematopoietic Stem Cell Transplantation
Source: Front Immunol. 2021 Dec 7;12:782780. doi: 10.3389/fimmu.2021.782780 (PMC8691729; doi:10.3389/fimmu.2021.782780)
Supplement: Supplementary file 1 [file DataSheet_1.docx]

Supplementary Material

# Supplementary Data

**1.1 Methods of whole-exome sequencing**

Genomic DNAs were extracted from peripheral blood leukocytes using a DNA blood kit (Qiagen, Hilten, Germany).

To perform whole exom sequencing, libraries were prepared according to the Agilent SureSelect Target Enrichment Kit preparation guide using SureSelect Human All Exon V4 (Agilent Technologies, Santa Clara, CA, USA). The libraries were sequenced with the Illumina HiSeq 2500 platform (Illumina, San Diego, CA, USA). Reads were aligned to the human reference genome GRCh37/hg19 using Burrows-Wheeler Aligner 0.7.10.

**1.2 Methods of Sanger sequencing**

After variant identified with WES, the variant was confirmed using Sanger sequencing method. PCR amplifications were done with primer set for exon9 of *MVK*, forward of 5’-GTGGTTTCCCCAGAGGATGG-3’ and reverse of 5’-TAGCTTCCGGGGGATTCTGA-3’. Amplified PCR products were sequenced with BigDye Terminator v3.1 using same primers at PCR and than electrophoresed in ABI3130*xl* Genetic Analyzer (Applied Biosystems, Foster city, CA, USA).

**1.3 Methods of real-time polymerase chain reaction**

To identify heterozygous exonic deletion of *MVK*, SYBR green quantitative PCR was performed using TB Green Premix Ex Taq (Takara Bio.inc, Shiga, JP) and MJ research PTC-200 Thermo Cycler (MJ research Inc. CA, USA). The control primer was for exon9 of *MVK,* which had heterozygous variant meaning normal dosage. Target primer set for exon11 of *MVK* was forward of 5’- TGCAGGTAACCTTGGGCTTT-3’ and reverse of 5’- GAACTGCAGCCCCCAGAATA-3’. Dosage analysis was carried out using MJ Opticon Monitorv3.1 (MJ research Inc. CA, USA).

# Supplementary Figures and Tables

## Supplementary Figures


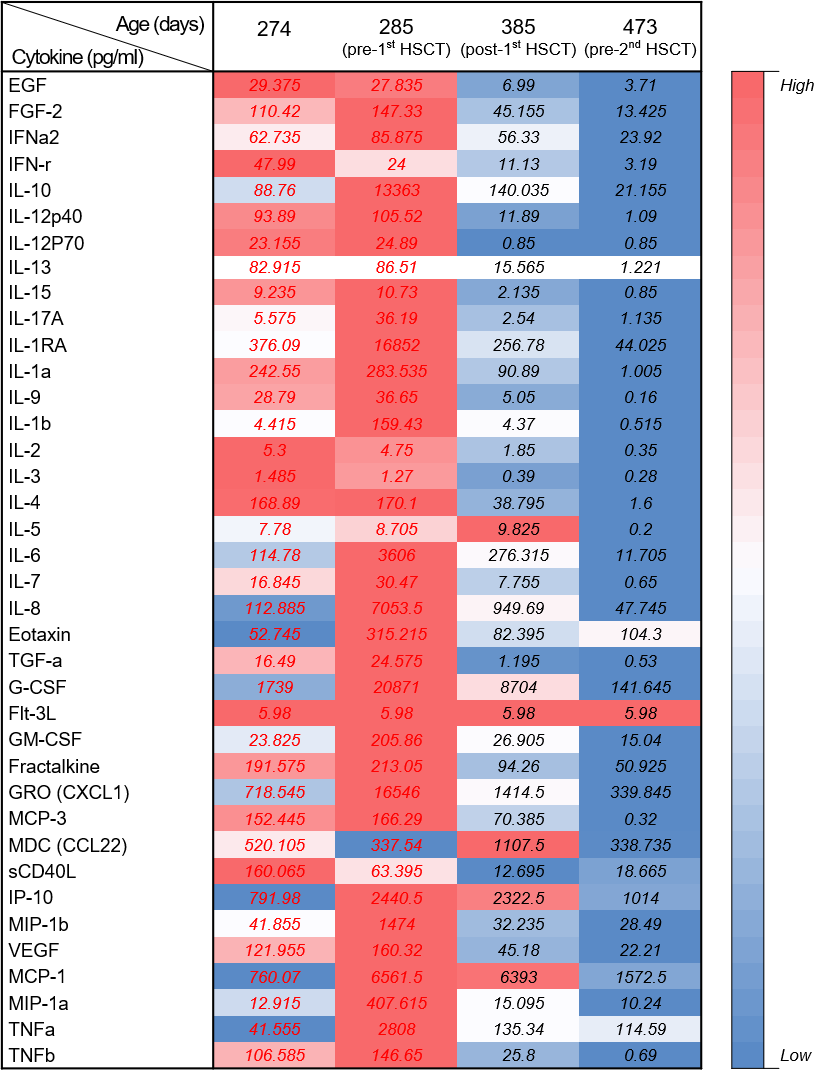


**Supplementary Figure 1.** Cytokine assay using the Millipore Human Cytokine/Chemokine Magnetic Bead Panel, along with the patient’s clinical course, expressed as a heatmap. The levels of most cytokines increased immediately after diagnosis, which peaked at the time of the 1^st^ HCT. Cytokine levels were markedly decreased after the 1^st^ HCT and reached a minimum at the time of the 2^nd^ HCT. HCT, hematopoietic stem cell transplantation.
